# Olive leaf extract-assisted green synthesis of cd nano complex: A combined experimental and theoretical study

**DOI:** 10.1371/journal.pone.0306040

**Published:** 2024-08-02

**Authors:** Mutairah S. Alshammari, Rania H. Taha, Nowarah J. Almutlq, Sabrein H. Mohamed

**Affiliations:** 1 Chemistry Department, College of Science, Jouf University, Sakaka, Saudi Arabia; 2 Department of Chemistry, Faculty of Science (Girls), Al-Azhar University, Nasr City, Cairo, Egypt; 3 Chemistry Department, Faculty of Science, Cairo University, Cairo, Egypt; Ahram Canadian University, EGYPT

## Abstract

Research in the synthesis of Schiff base ligands and their metal complexes using olive leaf extracts as a green reducing agent is an exciting area of study. In this research, a Schiff base ligand is created by combining 1-hydroxy-2-naphthaldehyde and amino-*N*-(4,6-dimethylpyrimidin-2-yl)-4-benzenesulfonamide. The synthetic Schiff base is then utilized for the production of a Cd(II) nano complex for the first time with olive leaf extracts serving as the green reducing agent. The extract is obtained by harvesting, drying, and grinding the olive leaves. Various analytical techniques, including ^1^H NMR, ^13^C NMR spectroscopy, scanning electron microscope (SEM), and conductivity studies, are employed to analyze the Schiff base and its Cd(II) complex. Quantum chemical calculations are also conducted to explore the different conformers of the Cd(II) complex and their stabilities, shedding light on the synthesis pathways of the Schiff base ligand and Cd(II) complex. Extensive DFT-based geometry optimizations and frequency calculations are carried out for 1-hydroxy-2-naphthaldehyde,amino-*N*-(4,6-dimethylpyrimidin-2-yl)-4-benzenesulfonamide, the Schiff base ligand, and the corresponding Cd(II) complex. Experimental and theoretical analyses confirm the presence of the azomethine (-HC = N-) group in the Schiff base and validate the formation of the Cd(II) complex in a 2:1 metal-to-ligand ratio through physicochemical characterization methods, highlighting the nanoscale structure of the complex. Combining thorough physicochemical investigations with molecular modeling simulations and the sustainable synthesis of metal complexes, valuable insights into their properties and potential applications in catalysis and drug delivery are obtained.

## Introduction

Schiff bases are formed when an aldehyde or ketone reacts with a primary amine When an aldehyde or ketone is combined with a primary amine [1, 2], resulting in a compound with an azomethine group containing HC = N bonds [3]. These ligands are essential in coordination chemistry for creating stable complexes with metal ions, first discovered by Hugo Schiff in 1864 [4, 5], Schiff base ligands play a crucial role [6, 7] based on their physicochemical characteristics [1]. They can be used in a variety of clinical [8], biological [4], and analytical branches [9].

Sulfonamides are a significant class of organo-sulfur compounds that belong to the sulfa medication family. Their main chemical structure includes the sulfonamide group (SO_2_NH) and an amino group (NH_2_). Sulfonamides are cost-effective, stable antibiotics widely used in veterinary medicine to treat and prevent bacterial infections in animals.

Olives have been historically valued for their food and medicinal properties. Olive leaf extract is effective in treating fever and malaria [10]. It contains substances with strong antibacterial effects [11, 12]. Olives also have anti-inflammatory and antioxidant effects. The olive plant can prevent HIV-1 infection and cell-to-cell transfer [13, 14]. The main active compounds in olive leaves are oleuropein, hydroxytyrosol, and tyrosol. Other active compounds include luteolin-7-glucoside, apigenin-7-glucoside, diosmetin-7-glucoside, caffeic acid, p-coumaric acid, vanillic acid, vanillin, luteolin, diosmetin, and rutin [15, 16].

This study explored the first-ever use of olive leaf extracts in a bio-reduction method to synthesize a stable cadmium (II) nano complex. Experimental and theoretical methods were employed to characterize the structure, contributing to existing knowledge on the biosynthesis of nano complexes using plant leaf extracts. The experimental toolbox included SEM imaging, conductivity tests, ^1^HNMR, and ^13^C NMR. Additionally, by simulating the relevant processes in the synthesis of the Schiff base ligand and the Cd(II) complex using quantum chemical simulations, we were able to discover different conformers for the Cd(II) complex and their corresponding stability profiles. Concerning 1-hydroxy-2-naphthaldehyde, amino-*N*-(4,6-dimethylpyrimidin-2-yl)-4-benzenesulfonamide, the Schiff base ligand, and the associated Cd(II) complex, we specifically carried out DFT-based extensive geometry optimization and frequency simulations.

## Methods

### Materials and instruments

Sulphadimidine, Cd(Cl)_2_.H_2_O, 1-hydroxy-2-naphthaldehyde, and ethyl alcohol were purchased and used without further purification (Aldrich, USA). The ^1^H NMR, and ^13^C NMR spectra (in DMSO-*d*_*6*_) were acquired on a 600MH_Z_ spectrometer without the use of an internal standard. With a 5–10 keV functioning Jeol 6310 (Jeol Instruments, Tokyo, Japan) piece of equipment, scanning electron microscopy (SEM) (gold coating, Edwards Sputter Coater, UK) research was conducted.

#### Collection of samples and extraction

Olive leaves were collected from different locations in Sakaka Gardens, Al-Jouf city, Saudi Arabia. The undamaged olive leaves were washed, dried, and ground into powder. 25 g of the powder was mixed with 100 mL of methanol in a conical flask and agitated for 48 hours. The mixture was filtered using Whatman No. 1 filter paper and then evaporated to create a nano complex with the extract.

### Synthesis of the free ligand H_2_L

**Fig 1:**
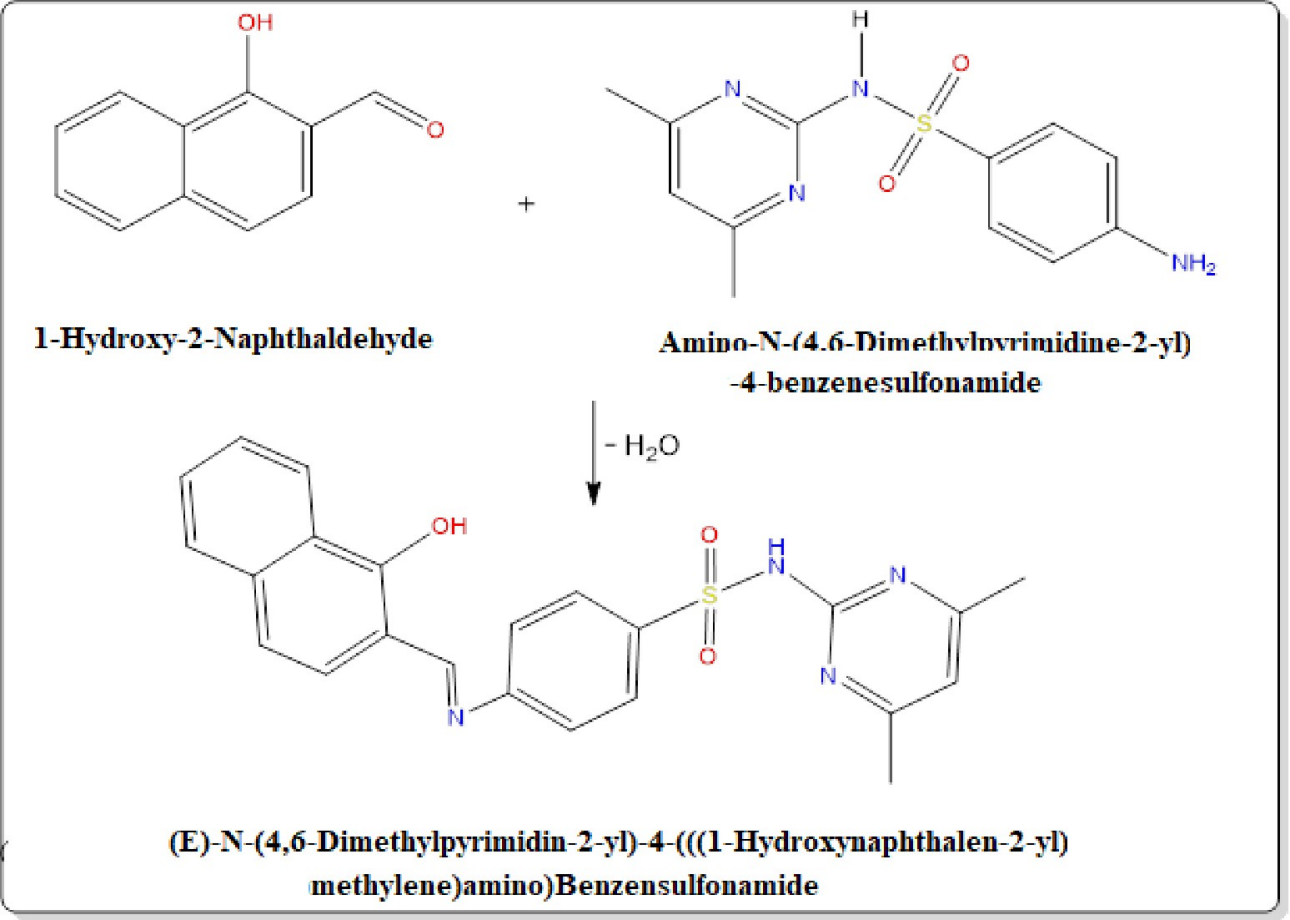
Schematic representation of the tetradentate ligand H_2_L.

The ligand was synthesized using a previously described procedure [17]. A solution of 1-hydroxy-2-naphthaldehyde (4.305 g, 0.025 mol) in ethanol was mixed with an ethanolic solution of sulphadimidine (6.958 g, 0.025 mol). The reaction mixture was cooled and then refluxed for two hours in a water bath. After filtration and washing, a dark yellow powder weighing 10.70 g was obtained with a 95% yield. The product was dried in a desiccator over anhydrous calcium chloride. The predicted structure of the ligand (**Fig** 1) and the stoichiometry are consistent.

### Biosynthesis of Cd nano complex

The metal complex was synthesized by mixing 0.0025 mol of the ligand H_2_L with 0.005 mol of Cd(Cl)_2_.H_2_O (1:2 molar ratio) in the presence of olive extract as an auxiliary agent. The mixture was ground using a pestle in an open mortar at room temperature. After allowing the mixture to solidify, the solid was filtered and crystallized twice in ethanol to obtain the Cd-nano complex as a light brown crystal. The isolated complex is insoluble in most organic solvents but soluble in DMF and DMSO and appears as a powder. It is stable in the air.

### Quantum chemical calculations

The reaction of the Schiff base ligand (**H**_**2**_**L**) from amino-N-(4,6-dimethylpyrimidin-2-yl)-4-benzenesulfonamide (ANB) and 1-hydroxy-2-naphthaldehyde (HN) was simulated using quantum chemical calculations. The procedure involves a full gradient minimization geometry optimization of HN, ANB, **H**_**2**_**L**, and a single water molecule. The optimized geometries showed positive harmonic vibrational frequencies and zero gradient norms. These calculations were carried out using the Gaussian16 software package [18] and the Becke three-parameter Lee-Yang-Parr hybrid functional (B3LYP) density functional theory (DFT) [19, 20]. All atoms [21] were subjected to the Def2TZVPP basis set and Grimme’s D3 dispersion correction [22].

For the condensation reaction: 1 HN + 1 ANB → 1 **H**_**2**_**L** + 1 H_2_O, the reaction energy (ΔE) was determined according to the following equation:

$$\Delta E=E(H_{2}L)+E(H_{2}O)‐E(HN)‐E(ANB)$$ (Eq 1)

Here, E(H_2_L), E(H_2_O), E(HN), and E(ANB) stood for the total electronic energies of **H**_**2**_**L**, H_2_O, HN, and ANB, respectively, including the dispersion energy adjustment. While accounting for zero-point energy and thermal adjustment for enthalpy and Gibbs free energy, the appropriate reaction enthalpy (H) and reaction free energy (G) were determined.

The interaction between the Schiff base ligand (**H**_**2**_**L**) and CdCl_2_ to create the Cd(II) complex (Cd_2_ **H**_**2**_**L**) was similarly simulated in a way comparable to the aforementioned condensation reaction and using the same theoretical methodology and degree of theory. Calculations were made for each reaction’s energy (E), enthalpy (H), and free energy (G). Based on projected preferred binding orientations and conformations, the first geometries for the Cd(II) complex were constructed. The Cd(II) complex now has four possible conformers. For each Cd(II) complex conformer, the reaction was simulated as 1 **H**_**2**_**L** + 2 CdCl_2_ → 1 Cd_2_ **H**_**2**_**L**, and the corresponding reaction energy (ΔE) was calculated as follows:

$$\Delta E=E(Cd_{2}\;H_{2}L)‐E(H_{2}L)‐2\;*\;E(CdCl_{2})$$ (Eq 2)

Here, E(Cd_2_ **H**_**2**_**L**), E(**H**_**2**_**L**), and E(CdCl_2_) denoted, respectively, the total electronic energies for each of the Cd(II) complex conformers, Cd_2_ **H**_**2**_**L**, **H**_**2**_**L**, and CdCl_2_. The matching reaction enthalpy (H) and reaction free energy (G) were calculated with similar thermal and zero-point energy corrections. Since there are many different Cd(II) complex conformers that could exist, Eq 2 produced distinct sets of E, H, and G values, each of which was unique to a given conformer.

## Results and discussion

### (*E*)-*N*-(4,6-dimethylpyrimidin-2-yl)-4-(((2-hydroxynaphthalen-1-yl)methylene)amino) benzenesulfonamide ligand (H_2_L) and its Cd nano metal

Elemental analysis results for the synthesized ligand and its nano Cd complex matched the expected calculations (Elemental Analysis for H_2_L free ligand: C, 63.87(63.50); H, 4.66(4.26); N, 12.95(12.80); O, 11.10(10.98); S, 7.41(732)). Additionally, the produced product is supposed to have a stoichiometry of 1:2 (ligand: metal). The metal complex has a molar conductance of 29.60 Ohm^-1^cm^2^mol^-1^ indicating it is non-electrolytic. The ligand and its nano complex were found to have melting points of 142°C, and >350°C respectively demonstrating the purity and stability of the compounds.

### ^1^H NMR spectra

**Fig 2:**
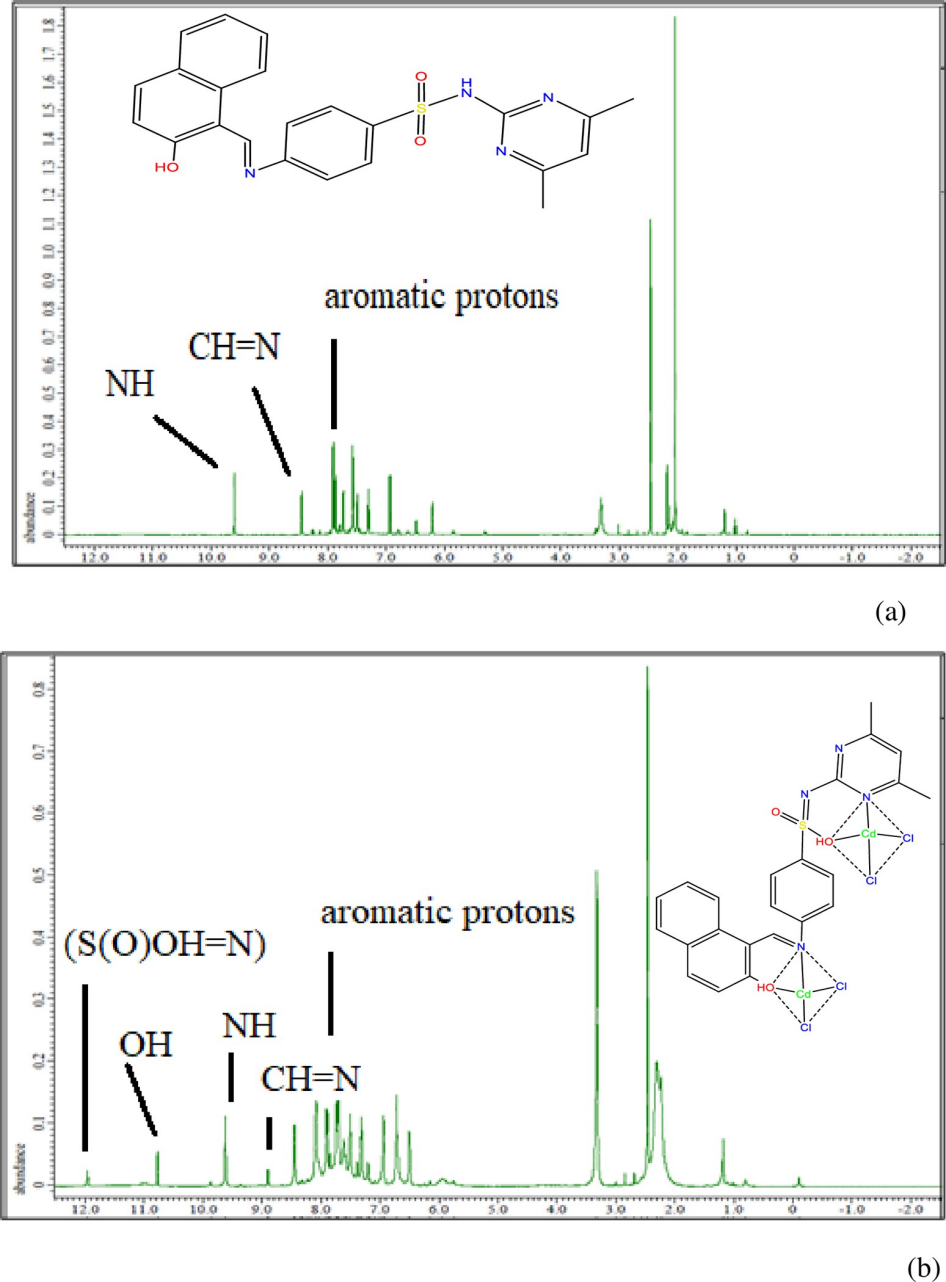
^1^H NMR spectra of (a): the free ligand **H**_**2**_**L** and (b): The Cd-nano complex.

The ^1^H NMR spectrum of the nano complex showed singlet signals at 9.48 and 8.29 ppm from NH and CH = N protons, and multiplet signals at 7.55–7.96 ppm induced from aromatic protons. A new peak appeared due to the enolization of the sulphonamide group(-SO_2_NH groups to the (S(O)OH = N)), indicating complexation. Comparison with the free ligand confirmed coordination with the metal atom. The OH group peak shifted to 10.79 ppm, indicating coordination and the azomethine group peak shifted to 8.90 ppm. Aromatic protons showed multiplet signals between 7.79 and 7.94 ppm, while OH protons remained unchanged (**Fig 2A and 2B**).

### ^13^C NMR spectra

**Fig 3:**
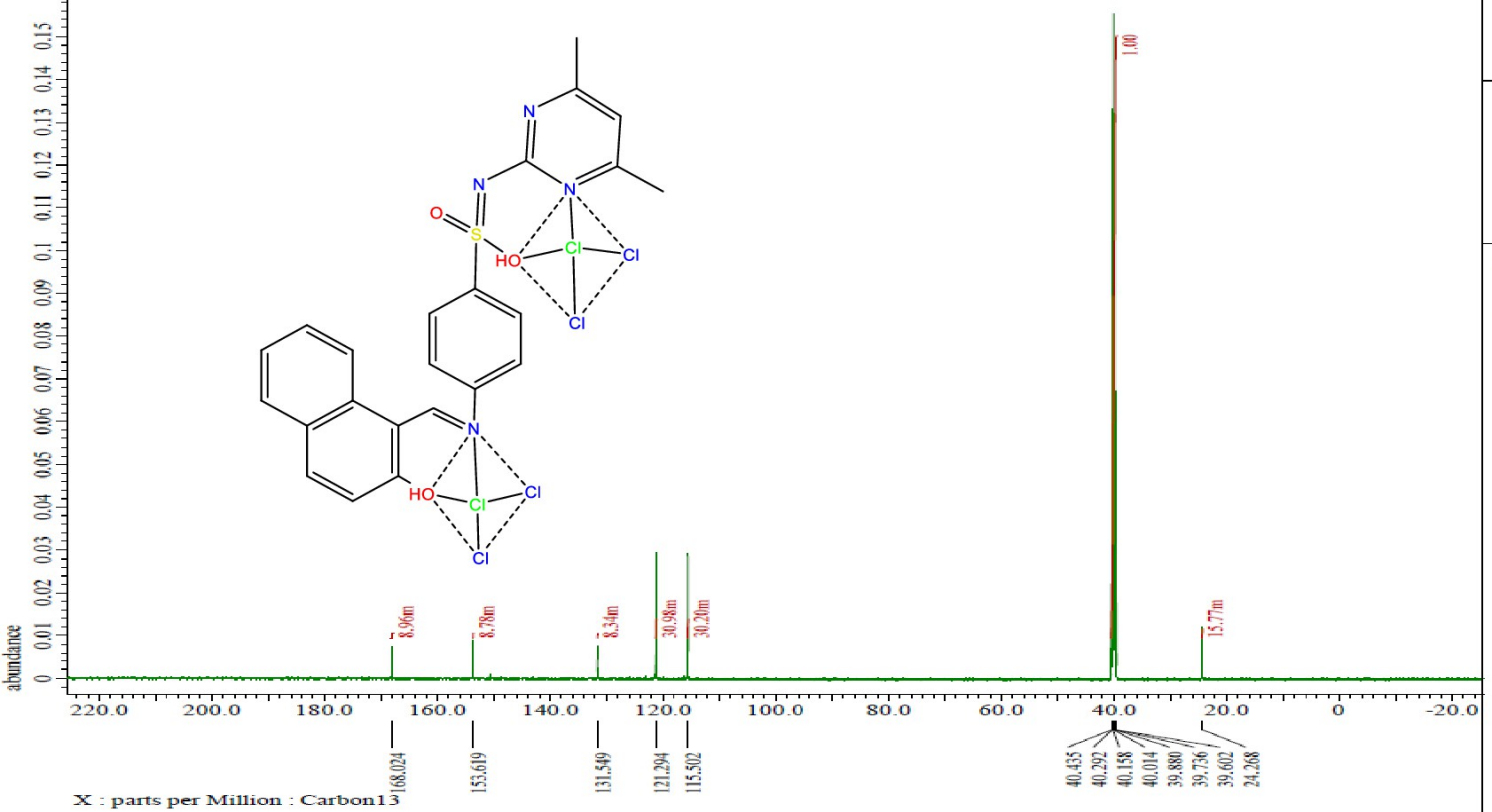
^13^C NMR spectrum of the Cd-nano complex.

The Cd-nano complex’s ^13^C NMR spectrum in DMSO-d6 was obtained (**Fig** 3). All identified carbon atoms undergo chemical shifts, which are shown in Table 1. Upon comparison it was found that only the carbon atom of the carbonyl group was altered, suggesting that the oxygen in this group is solely involved in complexation [23, 24].

**Table 1:** ^13^C chemical shifts (in ppm) for the free ligand H_2_L and Cd nano complex.

Carbon	^13^C chemical shift for Cd-nano complex	Assigned to
**1**	153.6	C of benzene ring
**2**	168.02	C of the carbonyl group
**3**	24.26	C of the methyl group
**4**	115.5	C of benzene ring
**5**	121.9	C of benzene ring
**6**	131.5	C of benzene ring attached to the NH group

### Conductivity measurements

The molar conductivities of 1.0 mmol/l Cd-nano complex solutions were measured at standard temperatures. The results support the coordination mechanism of nitrates with the metal cation and show that the metal complex is non-ionic, making it clear that the complex is a nonelectrolyte.

### SEM images

**Fig 4:**
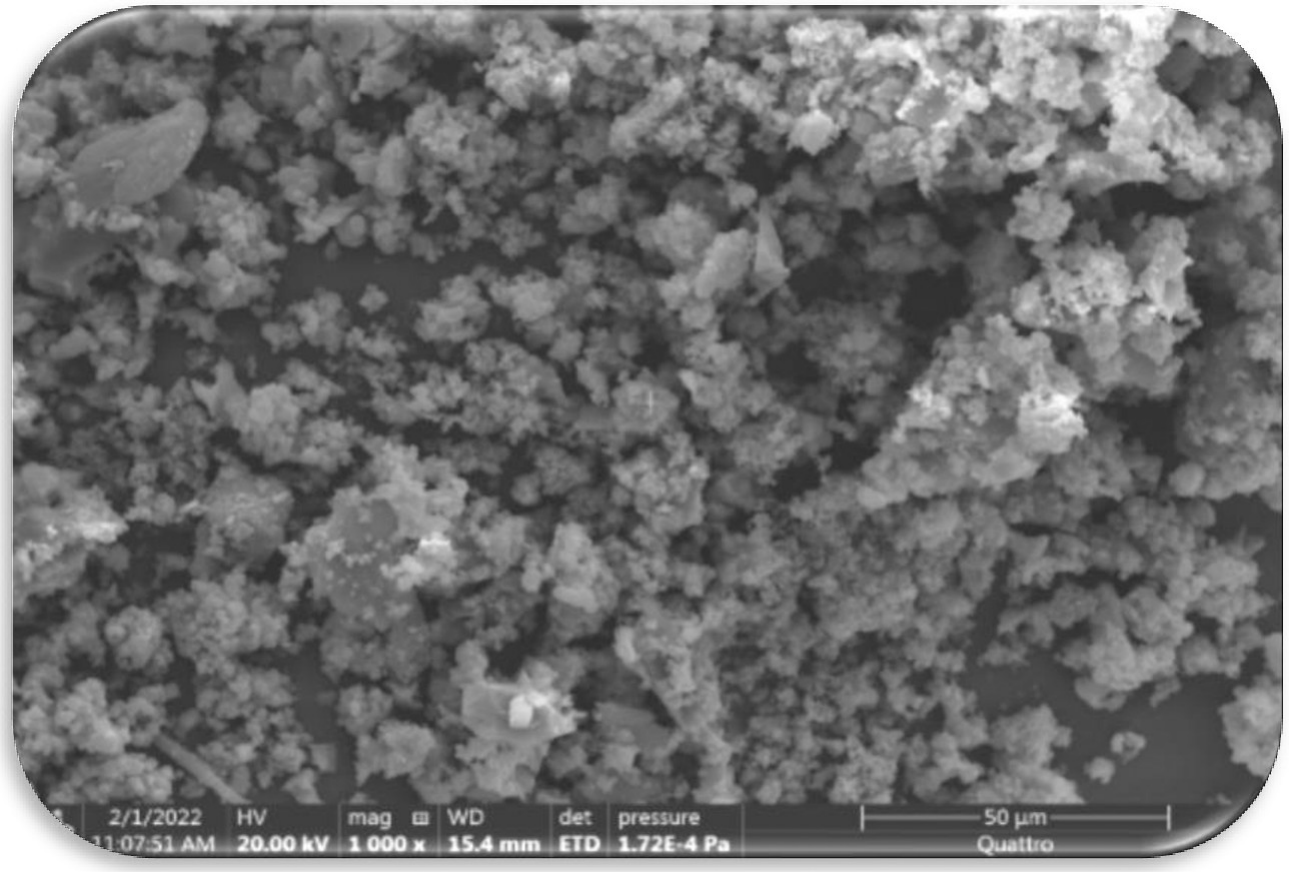
SEM image of Cd-nano complex.

SEM techniques were used to analyze the nano particle’s microstructure, as depicted in **Fig 4**. The particles were identified as cubic and spherical, with an estimated size of 4–55 nm, based on this photograph. This study provides a novel approach to biosynthesizing Cd-nano complexes, distinct from other nanoparticle creation methods.

### The X-ray diffraction (XRD) pattern of the prepared sample of Cd nanoparticle

**Fig 5:**
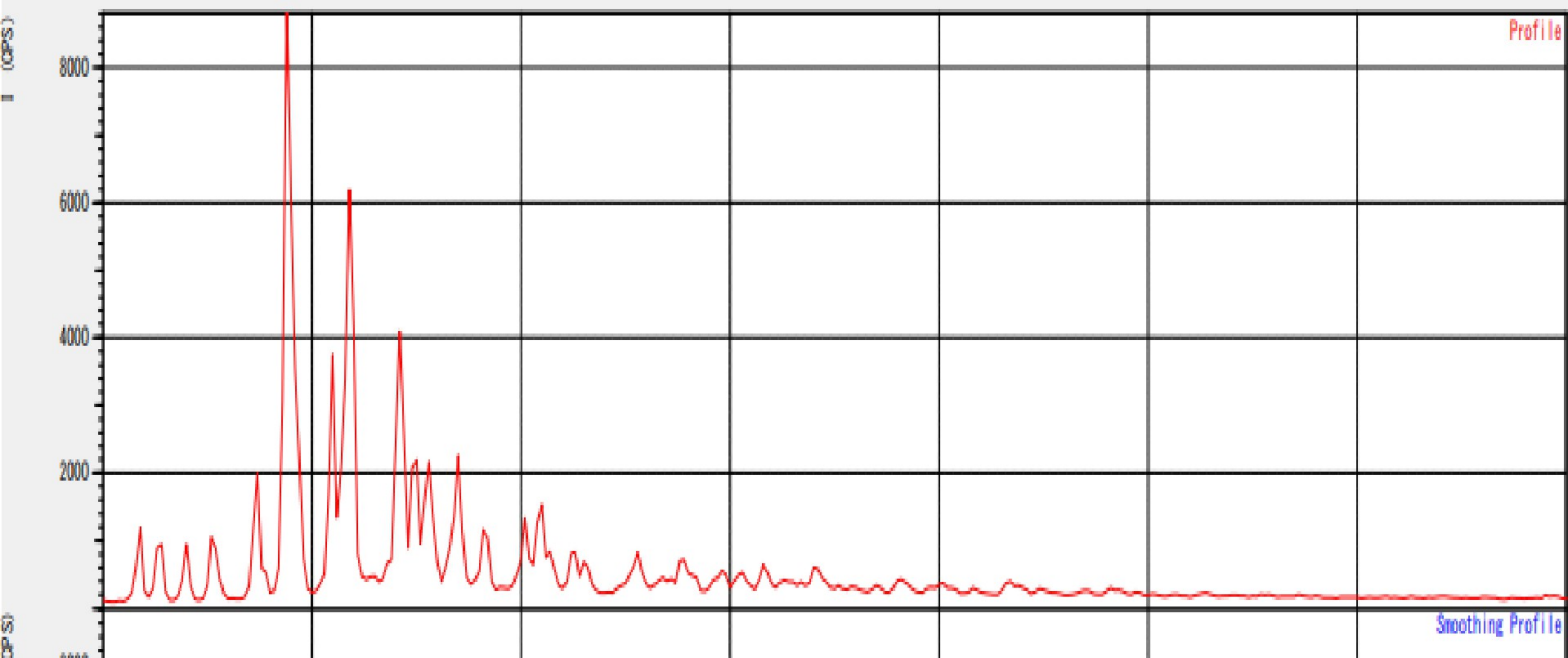
X-ray diffractogram of Cd-nanocomplex.

The X-ray diffraction (XRD) pattern of the manufactured Cd nanoparticles sample was obtained and shown in **Fig** 5 using CuK radiation (λ = 1.5406), 40 kV- 40mA, and 2θ scanning mode. This analysis aimed to confirm the crystallinity of the Cd-nano compound. The data collection range was between 10 and 80 degrees. A comparison was made between the experimental diffractogram (**Fig** 5), Cd file No. 04–0783, and the JCPDS standard powder diffraction card. Four peaks at 2θ values of 45.30°, 55.50°, 68.70°, and 79.80° in the experimental diffractogram were identified as originating from Cd metal.

The average crystalline size D of the Cd nanoparticles was determined by calculating from the diffractogram using the Debye-Scherrer formula, D = 0.9/Cos, where Cos represents the full width at half maximum (FWHM) of a peak and is the wavelength of the X-rays used for diffraction [25]. The FWHM was obtained by fitting a Gaussian function to each of the four peaks. The fitted Gaussian curve was then utilized to calculate the FWHM of the peak.

**Fig 6:**
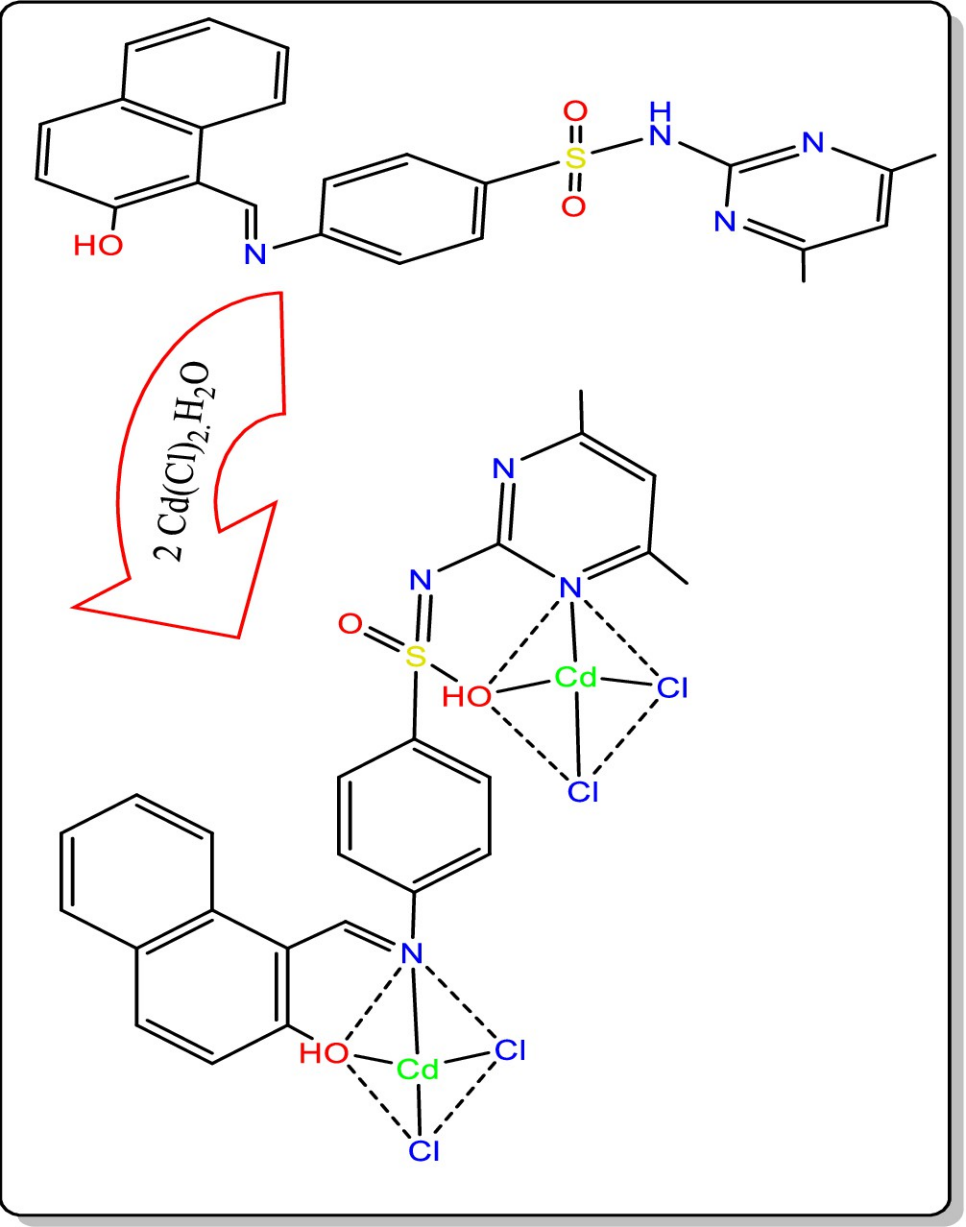
Suggested structure of the Cd nano metal complex.

The results collected for the complex under investigation show a correlation that provides information on the suggested structure of the complex, as depicted in **Fig** 6.

### Quantum chemical calculations

**Fig 7:**
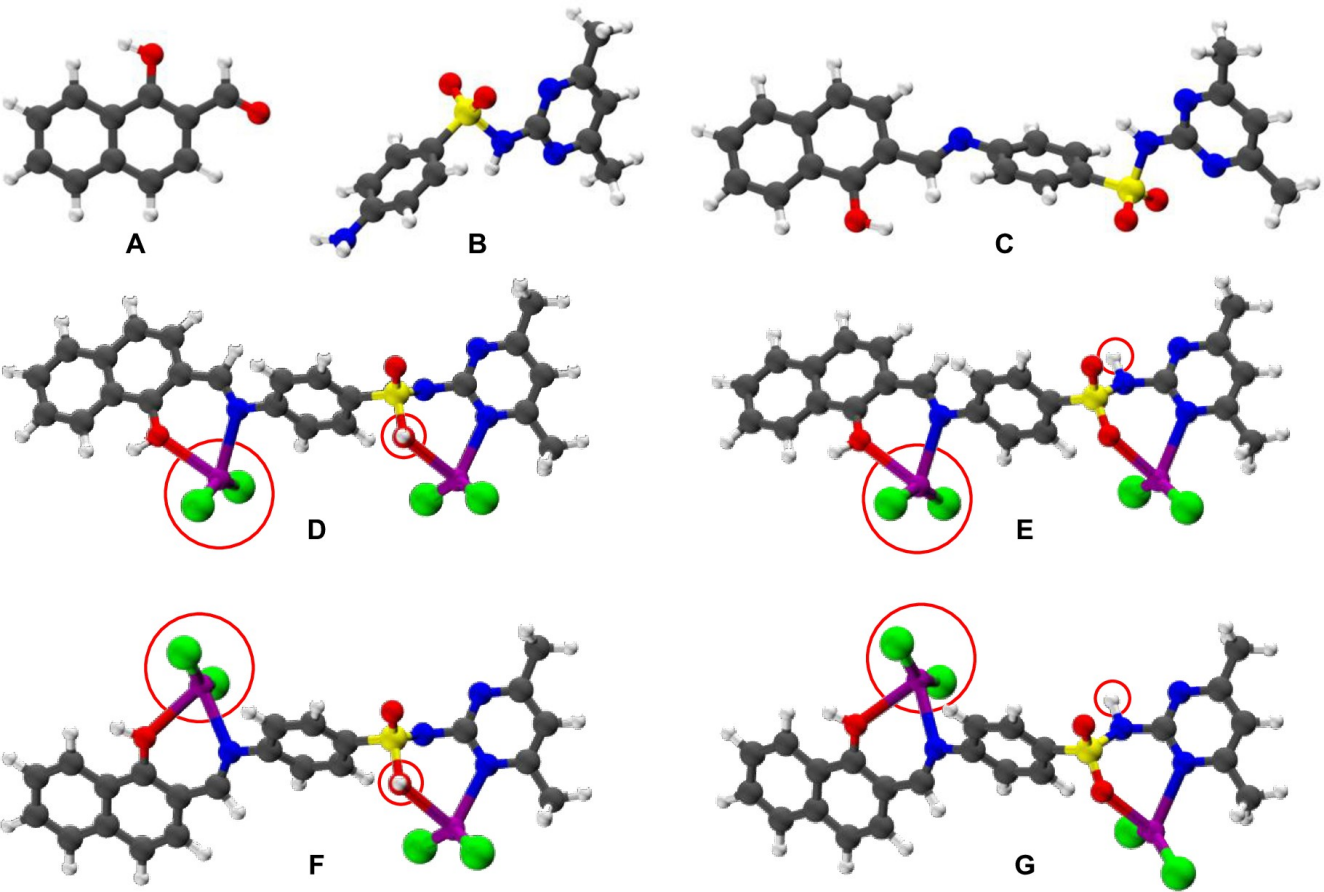
Optimized structures of 1-hydroxy-2-naphthaldehyde (A), amino-N-(4,6-dimethylpyrimidin-2-yl)-4-benzenesulfonamide (B), the Schiff base ligand **H**_**2**_**L** (C), and Cd(II) complex conformers (D-G). Conformers D and F exhibit a zwitterionic configuration, while conformers E and G feature a neutral arrangement. Additionally, D and E conformers adopt cis configurations, whereas F and G conformers adopt trans configurations.

**Fig** 7 displays the optimized structures of the Schiff base ligand (**H**_**2**_**L**), 1-hydroxy-2-naphthaldehyde (HN), amino-N-(4,6-dimethylpyrimidin-2-yl)-4-benzenesulfonamide (ANB), and the Cd(II) complex (Cd_2_ **H**_**2**_**L**) conformers. The condensation reaction that forms the Schiff base ligand (**H**_**2**_**L**) has positive values for E, H, and G, indicating that the reaction is endothermic and not spontaneous (Table 2). Despite these small values, the reaction is still considered viable and responsive to external stimuli. In contrast, the production of the Cd(II) complex is exothermic and spontaneous, with significantly negative and moderately high E, H, and G values, indicating a favorable reaction leading to the spontaneous formation of a strong and stable complex, the Cd(II) complex.

**Table 2:** Reaction energy (ΔE), enthalpy (ΔH), and free energy (ΔG) for the condensation reaction (Eq 1) and complexation reaction (Eq 2).

Reaction	ΔE	ΔH	ΔG
	(kcal/ mol)	(kcal/ mol)	(kcal/ mol)
**Ligand**	+3.85	+3.25	+4.43
**Complex1 (D)**	-23.56	-21.47	-6.76
**Complex2 (E)**	-45.10	-42.19	-26.74
**Complex3 (F)**	-25.36	-23.20	-7.89
**Complex4 (G)**	-47.36	-44.37	-28.48

The sequence of stability is revealed as D F E G, as shown in **Fig** 7, by comparing the E, H, and G values for different conformers of Cd(II) complexes (distinguished principally by differences in electronic energy). The least stable conformers, D and F, have a zwitterionic configuration, which is made up of a positively charged connected oxygen (OH) atom and a positively charged nitrogen atom. The most stable conformers, E and G, on the other hand, take on a neutral structure. According to an analysis of the trends in energy, enthalpy, and free energy, the neutral complex structures are more stable than their corresponding zwitterionic counterparts in this situation. This difference could be explained by hydrogen bonding interactions between the NH group and the electronegative O and N atoms around it in neutral configurations.

Additionally, complex G’s superior stability over complex E can be due to the latter’s lessened repulsion of the choline atoms. The location of two CdCl_2_ groups—cis in the case of complex E and trans in the case of complex G—is the cause of this disparity. Therefore, it is anticipated that the neutral trans configuration will predominately be present in the Cd complex, with modest amounts of the cis configuration.

Based on the obtained, the future work will be the calculation of the charge density of the outermost molecular orbitals along with the molecular electrostatic potential (MEP) map.

## Conclusion

Extensive DFT-based geometry optimizations and frequency calculations were conducted for 1-hydroxy-2-naphthaldehyde and amino-N-(4,6-dimethylpyrimidin-2-yl)-4-benzenesulfon- amide, a Schiff base ligand, and their Cd(II) complex. The results confirmed the presence of the azomethine group in the Schiff base. Physicochemical characterization confirmed the formation of the Cd(II) complex in a 2:1 metal-to-ligand ratio, highlighting its nanoscaled structure. The analysis, along with molecular modeling simulations and sustainable biosynthesis, provides valuable insights into the complex’s properties and potential applications in catalysis and drug delivery. Future quantum chemical calculations will include determining the charge density of the outermost molecular orbitals and generating a molecular electrostatic potential (MEP) map.
